# Comparison of Predictability Using Barrett Universal II and SRK/T Formulas according to Keratometry

**DOI:** 10.1155/2020/7625725

**Published:** 2020-06-19

**Authors:** Kei Iijima, Kazutaka Kamiya, Yoshihiko Iida, Nobuyuki Shoji

**Affiliations:** ^1^Department of Ophthalmology, School of Medicine, Kitasato University, Tokyo, Japan; ^2^Visual Physiology, School of Allied Health Sciences, Kitasato University, Tokyo, Japan

## Abstract

**Purpose:**

To compare the predictability of intraocular lens (IOL) power calculation using the Barrett Universal II and the SRK/T formulas, according to the keratometry.

**Methods:**

We retrospectively reviewed the clinical charts of 335 consecutive eyes undergoing standard cataract surgery. IOL power calculations were performed using the Barrett Universal II and the SRK/T formulas. We compared the prediction error, the absolute error, and the percentages within ±0.25, ±0.5, and ±1.0 D of the targeted refraction, 1 month postoperatively, and also investigated the relationship of these outcomes with the keratometric readings, using the two formulas.

**Results:**

The prediction error using the SRK/T formula was significantly more myopic than that using the Barrett Universal II formula (the paired *t*-test, *p* < 0.001). The absolute error using the SRK/T formula was significantly larger than that using the Barrett Universal II formula (*p*=0.006). We found a significant correlation between the prediction error and the keratometric readings using the SRK/T formula (Pearson correlation coefficient, *r* = −0.522, *p* < 0.001), but there was no significant correlation between them using the Barrett Universal II formula (*r* = −0.031, *p*=0.576).

**Conclusions:**

The Barrett Universal II formula provides a better predictability of IOL power calculation and is less susceptible to the effect of the corneal shape, than the SRK/T formula. The Barrett Universal formula, instead of the SRK/T formula, may be clinically helpful for improving the refractive accuracy, especially in eyes with steep or flat corneas.

## 1. Introduction

Cataract surgery has been widely recognized as one of the refractive surgeries to correct refractive errors as much as possible. Although the refractive outcomes of cataract surgery have much improved in recent years, it is still challenging to obtain good outcomes in eyes having abnormal axial length and/or corneal shape in daily practice. According to the clinical survey of the Japanese Society of Cataract and Refractive Surgery in 2018, [1] the SRK/T formula is currently still the most used formula for intraocular lens (IOL) power calculation in Japan. [2] Recently, it has been demonstrated that the Barrett Universal II formula provided a higher predictability than the SRK/T formula, especially in eyes with long axial length [3–7].

However, to date, there have only been a few studies on the predictability of the IOL power calculation using the two major formulas, according to the keratometric readings [8–10]. Moreover, the detailed relationship between the predictability outcomes and the keratometry has so far not been elucidated using the two major formulas. It may give us intrinsic insights on the effect of the corneal shape on the refractive accuracy in daily practice. The goal of the current study is to retrospectively compare the refractive accuracy of IOL power calculation using the Barrett Universal II and the SRK/T formulas, according to the keratometric readings.

## 2. Materials and Methods

### 2.1. Study Population

The study protocol was registered with the University Hospital Medical Information Network Clinical Trial Registry (000036371). This retrospective study comprised a total of 335 eyes of 335 consecutive patients (135 men and 200 women, mean age ± standard deviation: 70.1 ± 9.3 years), who underwent standard phacoemulsification with nontoric monofocal IOL implantation, between July 2005 and January 2018 at the Kitasato University Hospital, and who completed a 1-month follow-up. Eyes with postoperative best corrected visual acuity of ≥0.15 logMAR, eyes with any history of ocular surgery, ocular trauma, or other concomitant eye diseases, and eyes developing any intraoperative or postoperative complications that could affect refractive outcomes were excluded from the study. Only one eye was randomly chosen from each patient for statistical analysis, when bilateral cataract surgery was performed. This retrospective review of the data was approved by the Institutional Review Board at the Kitasato University (B17-292) and followed the tenets of the Declaration of Helsinki. Our Institutional Review Board waived the requirement for informed consent for this retrospective study.

### 2.2. Cataract Surgical Procedures

For cataract surgery, three experienced surgeons conducted standard phacoemulsification, followed by IOL implantation. The surgical technique consisted of a capsulorhexis, nucleus and cortex extraction, and IOL implantation, through a 2.8-mm temporal corneal incision. Nontoric monofocal IOLs (AQ-110NV, STAAR Surgical, Chiba, Japan, and PU-6A, Kowa, Aichi, Japan) were implanted in 248 and 87 eyes, respectively. Postoperatively, steroidal, antibiotic, and bromfenac sodium medications were topically administered for 1 month, the dose being reduced gradually thereafter.

### 2.3. Assessment of Prediction Error and Absolute Error

IOL power calculations were performed with the Barrett Universal II formula and SRK/T formula, using axial length, keratometric readings (for both formulas), and anterior chamber depth (only for Barrett Universal II formula), measured with a partial coherence interferometer (IOL Master 500TM, Carl Zeiss Meditec, Jena, Germany). We optimized A-constants for each IOL power calculation by using the instrument's built-in software. The prediction errors defined by subtracting the predicted manifest spherical equivalent refraction from the manifest spherical equivalent 1 month postoperatively, these absolute values, and the percentages of eyes within ±0.25, ±0.5 and ± 1.0 D of the targeted refraction were calculated.

Based on the preoperative mean keratometric readings, we created the three subgroups: flat keratometry (<42 D; flat K), normal keratometry (42 D≤, <47 D; normal K), and steep keratometry (47 D≤; steep K) groups. We assessed the relationship between the prediction error and the mean keratometry, in order to clarify the effect of the keratometry on the refractive accuracy using the two IOL formulas.

### 2.4. Statistical Analysis

We conducted statistical analyses by using commercially available statistical software (BellCurve for Excel, Social Survey Research Information Co, Ltd., Tokyo, Japan). Since we confirmed normal distributions of the data using the Kolmogorov–Smirnov test, the paired *t*-test was used to compare the prediction errors when using the two IOL power calculation formulas. Fisher's exact test was used to compare the percentages of eyes within ±0.25, ±0.5 and ± 1.0 D of the targeted correction. The results are expressed as mean ± standard deviation, and a value of *p* < 0.05 was considered statistically significant.

## 3. Results

Table 1 shows the preoperative demographics of the study population.  Table 2 shows the prediction error and the absolute error of the targeted refraction, retrospectively, when using the Barrett Universal II and the SRK/T formulas. The prediction error (−0.10 ± 0.53 D) using the SRK/T formula was significantly more myopic than that (0.04 ± 0.42 D) using the Barrett Universal II formula (the paired *t*-test, *p* < 0.001). The absolute error (0.38 ± 0.38 D) using the SRK/T formula was significantly larger than that (0.33 ± 0.27 D) using the Barrett Universal II formula (*p*=0.006).  Table 3 shows the percentages within ±0.25, ±0.5 and ± 1.0 D of the targeted refraction. The percentage within ±1.0 D using the Barrett Universal II formula was significantly higher than that when using the SRK/T formula (Fisher's exact test, *p*=0.026), but there were no significant differences in the percentages within ±0.25 and ± 0.5 D using the two formulas (*p*=0.589 and *p*=0.148, respectively).

**Figure 1:**
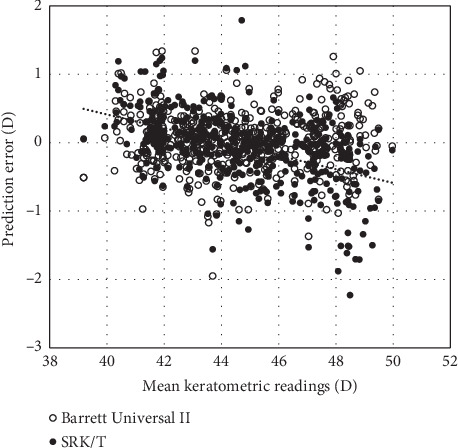
A graph showing correlations between the prediction error and the mean keratometry (Pearson correlation coefficient, *r* = −0.031, *p*=0.576 for the Barrett Universal II formula, and *r* = −0.522, *p* < 0.001 for the SRK/T formula).

We found a significant negative correlation between the prediction error and the keratometric readings using the SRK/T formula (Pearson correlation coefficient, *r* = −0.522, *p* < 0.001), but no significant correlation between them using the Barrett Universal II formula (*r* = −0.031, *p*=0.576) (Figure 1).

According to the keratometric readings, we found no significant differences in the prediction error using the two formulas in the normal K group (the paired *t*-test, *p*=0.182). On the other hand, the prediction error using the SRK/T formula was significantly more hyperopic than that using the Barrett Universal II formula in the flat K group (*p* < 0.001) and significantly more myopic than that using the Barrett Universal II formula in the steep K group (*p* < 0.001) (Table 2). However, we found no significant difference in the absolute error using the two formulas in the flat K or normal K group (*p*=0.334, *p*=0.761). On the other hand, the absolute error using the SRK/T formula was significantly larger than that using the Barrett Universal II formula in the steep K group (*p*=0.003) (Table 2).

## 4. Discussion

In the present study, our results showed that the use of the Barrett Universal II formula provided an overall higher predictability of IOL power calculation compared to the use of the SRK/T formula, in terms of the prediction error, the absolute error, and the percentages of eyes within ±0.25, ±0.5, and ±1.0 D of the targeted refraction, in the whole population, although there were no significant differences in the percentages within ±0.25 and ±0.5 D using the two formulas. Our results also showed that there was a significant correlation between the prediction error and the keratometric readings when using the SRK/T formula, but no significant correlation between them when using the Barrett Universal II formula.

Table 4 summarizes previous studies on the predictability of IOL power calculation in eyes having steep and flat corneas, respectively. So far only a few studies have been done on the effect of the corneal shape on the predictability of IOL power calculation. Olsen et al. [8] found a significant negative correlation of the prediction error with the keratometric readings (*r* = −0.23, *p* < 0.0001), when the SRK/T formula was used. Faramarzi et al. [9] demonstrated that the prediction error was −0.06 ± 0.52 D in eyes with a keratometry >46 D using the SRK/T formula, but that the sample size was limited (*n* = 45). Reitblat et al. [10] showed that myopic refractive errors (−0.31 ± 0.54 D) were found in eyes with a keratometry >46 D, but hyperopic errors (0.16 ± 0.31 D) were noted in eyes with a keratometry <42 D, when the SRK/T formula was used, and that the prediction error was −0.04 ± 0.45 D and −0.07 ± 0.26 D, in eyes with a keratometry >46 D and <42 D, respectively, both of which were not significantly different from zero, when the Barrett Universal II formula was used. Their previous findings are in accordance with our current findings in terms of the prediction error. Based on our findings, it is suggested that the SRK/T formula is susceptible to the effect of the corneal shape, whereas the Barrett Universal II formula is not susceptible to the effect of the corneal shape. Melles et al. [7] also showed that the SRK/T formula was adversely affected by eyes that have flat or steep keratometry, but that the Barrett Universal II formula tended to have the least bias of the formulas as measured by prediction error with variations in keratometry. We should be aware that adequate adjustment of the targeted correction is required when using the SRK/T formula, but not necessarily required when using the Barrett Universal II formula. We assume that the Barrett Universal II formula may be better than the SRK/T formula, especially not only in eyes with long axial length but also in eyes having steep or flat corneas, in order to further improve the refractive accuracy in such eyes, although both formulas provide excellent predictability in eyes having normal corneas.

There are at least the three limitations to this study. First, we included two IOL models for the assessment of the predictability in this study. However, we found no significant differences in the refractive outcomes between the two IOL models, when using the Barrett Universal II formula or the SRK/T formula. Accordingly, we assume that the effect of the IOL model on the outcomes was minimum and clinically negligible, in this case series. Second, we assessed the 1-month postoperative data, when the refraction was considered stable, which is the same as many published studies on the predictability of modern cataract surgery, although we accept that 3-month postoperative data would be ideal to determine the accurate refraction [11]. Third, the study was conducted in a retrospective fashion. A prospective study in a cohort of another population is still necessary to confirm the authenticity of our results.

In conclusion, our results may support the view that the Barrett Universal II formula provides a higher predictability of the IOL power calculation than the SRK/T formula and that the former formula is less susceptible to the preoperative keratometric readings than the latter formula. We believe that this information may be clinically helpful for understanding the properties of the two major IOL power calculation formulas, especially in eyes with steep or flat corneas.

## Figures and Tables

**Table 1:** The preoperative demographics of the study population undergoing cataract surgery.

	Mean ± standard deviation (range)
Number of eyes	335
Age	70.1 ± 9.3 years (29 to 89 years)
Male: female	135 : 200
logMAR CDVA	0.15 ± 0.25 (−0.08 to 1.40)
Mean keratometric readings	44.84 ± 2.50 D (39.19 to 49.90 D)
Axial length	24.05 ± 2.41 mm (20.48 to 31.11 mm)

logMAR = logarithm of the minimal angle of resolution; CDVA = corrected distance visual acuity; D = diopter.

**Table 2:** The refractive error of the targeted refraction using the Barrett Universal II and the SRK/T formulas.

Keratometry	Number of eyes	Refractive error	Barrett Universal II	SRK/T	*p* value
Entire	335	Prediction error (D)	0.04 ± 0.42 (−1.37 to 1.32)	−0.10 ± 0.53 (−2.75 to 1.19)	<0.001
		Absolute error (D)	0.33 ± 0.27 (0.00 to 1.37)	0.38 ± 0.38 (0.00 to 2.75)	0.006
		Median absolute error (D)	0.25	0.30	

Flat K group	69	Prediction error (D)	0.15 ± 0.42 (−0.65 to 1.32)	0.27 ± 0.40 (−0.63 to 1.19)	<0.001
		Absolute error (D)	0.35 ± 0.27 (0.01 to 1.32)	0.37 ± 0.30 (0.00 to 1.19)	0.334
		Median absolute error (D)	0.29	0.32	

Normal K group	180	Prediction error (D)	−0.02 ± 0.39 (−1.08 to 1.06)	−0.05 ± 0.39 (−1.15 to 1.09)	0.138
		Absolute error (D)	0.29 ± 0.24 (0.00 to 1.08)	0.30 ± 0.25 (0.00 to 1.08)	0.925
		Median absolute error (D)	0.23	0.23	

Steep K group	86	Prediction error (D)	0.08 ± 0.49 (−1.37 to 1.26)	−0.49 ± 0.63 (−2.75 to 0.66)	<0.001
		Absolute error (D)	0.38 ± 0.31 (0.01 to 1.37)	0.57 ± 0.56 (0.00 to 2.75)	0.003
		Median absolute error (D)	0.27	0.37	

K = keratometric readings; D = diopter.

**Table 3:** The percentages within ±0.25, ±0.5, and ± 1.0 D of the targeted refraction using the Barrett Universal II and the SRK/T formulas.

Keratometry	Within ± 0.25 D (%)			Within ± 0.5 D (%)			Within ± 1.0 D (%)		
	Barrett Universal II	SRK/T	*p* value	Barrett Universal II	SRK/T	*p* value	Barrett Universal II	SRK/T	*p* value
Entire	78	74	0.589	79	73	0.148	97	93	0.026
Flat K group	48	41	0.493	74	70	0.706	96	94	1.000
Normal K group	52	53	0.916	84	81	0.579	98	98	1.000
Steep K group	49	43	0.541	71	60	0.199	97	84	0.009

K = keratometric readings; D = diopter.

**Table 4:** Summary of previous studies on the predictability of intraocular lens power calculation in eyes with steep and flat corneas.

Author	Number of eyes	Mean keratometric readings (D)	IOL calculation formula	Prediction error (D)	Within ± 0.5 D (%)	Within ± 1.0 D (%)
Faramarzi et al [9]	45	46≦	SRK/T	−0.06 ± 0.52	78	89
			Holladay1	0.21 ± 0.51	64	96
			Haigis	0.16 ± 0.55	64	93
			Hoffer Q	0.36 ± 0.51	58	91

Reitblat et al [10]	79	46<	Barrett Universal II	−0.04 ± 0.45	76	96
			SRK/T	−0.31 ± 0.54	61	91
			Hill-RBF	−0.17 ± 0.35	83	98
			Hoffer Q	0.18 ± 0.57	70	92
			Holladay1	−0.06 ± 0.52	70	94
			Holladay2	−0.04 ± 0.51	73	96
			Olsen-A	0.07 ± 0.41	78	99
			Olsen-C	0.18 ± 0.41	68	99
			Haigis	0.17 ± 0.42	71	97
	92	<42	Barrett Universal II	−0.07 ± 0.26	97	100
			SRK/T	0.16 ± 0.31	86	100
			Hill-RBF	−0.14 ± 0.26	88	100
			Hoffer Q	−0.22 ± 0.68	80	96
			Holladay1	0.04 ± 0.39	83	99
			Holladay2	−0.02 ± 0.35	86	99
			Olsen-A	−0.06 ± 0.31	91	98
			Olsen-C	−0.17 ± 0.33	90	99
			Haigis	−0.31 ± 0.31	75	97

Current	86	47≦	Barrett Universal II	0.08 ± 0.49	71	97
			SRK/T	−0.49 ± 0.63	60	84
	69	<42	Barrett Universal II	0.15 ± 0.42	74	96
			SRK/T	0.27 ± 0.40	70	94

## Data Availability

The data that support the findings of this study are available from the corresponding author upon reasonable request.
